# Metabolic profiling reveals local and systemic responses of host plants to nematode parasitism

**DOI:** 10.1111/j.1365-313X.2010.04217.x

**Published:** 2010-05-11

**Authors:** Julia Hofmann, Abd El Naser El Ashry, Shahbaz Anwar, Alexander Erban, Joachim Kopka, Florian Grundler

**Affiliations:** 1 Department of Applied Plant Sciences and Plant Biotechnology, Institute of Plant Protection, BOKU – University of Natural Resources and Applied Life SciencesVienna, Peter Jordan-Straße 82, A-1190 Vienna, Austria; 2 Department of Prof. L. Willmitzer, Max Planck Institute for Molecular Plant Physiology (MPIMP)Am Mühlenberg 1, D-14476 Potsdam-Golm, Germany

**Keywords:** *Heterodera schachtii*, metabolic profiling, systemic effects, syncytia, nematode

## Abstract

The plant parasitic beet cyst nematode *Heterodera schachtii* induces syncytial feeding structures in Arabidopsis roots. The feeding structures form strong sink tissues that have been suggested to be metabolically highly active. In the present study, metabolic profiling and gene targeted expression analyses were performed in order to study the local and systemic effects of nematode infection on the plant host. The results showed increased levels of many amino acids and phosphorylated metabolites in syncytia, as well as high accumulation of specific sugars such as 1-kestose that do not accumulate naturally in Arabidopsis roots. A correlation-based network analysis revealed highly activated and coordinated metabolism in syncytia compared to non-infected control roots. An integrated analysis of the central primary metabolism showed a clear coherence of metabolite and transcript levels, indicating transcriptional regulation of specific pathways. Furthermore, systemic effects of nematode infection were demonstrated by correlation-based network analysis as well as independent component analysis. 1-kestose, raffinose, α,α-trehalose and three non-identified analytes showed clear systemic accumulation, indicating future potential for diagnostic and detailed metabolic analyses. Our studies open the door towards understanding the complex remodelling of plant metabolism in favour of the parasitizing nematode.

## Introduction

Obligate plant parasites possess multiple strategies to exploit their hosts. Among these, sedentary endoparasitic cyst nematodes have developed a highly specific interaction with susceptible plants. In order to improve the current knowledge about this pathosystem, the parasitic interaction between the beet cyst nematode *Heterodera schachtii* and the host plant *Arabidopsis thaliana* has been established as a well-defined and highly versatile research model with respect to available methodology (Sijmons *et al.*, 1991). Mobile second-stage juveniles (J2) invade plant roots and move intracellularly into the central cylinder where they pierce a single plant cell with their stylets. Nematode secretions provoke severe morphological reorganization of the selected initial feeding cell, such as nucleus enlargement, proliferation of mitochondria and plastids, cytoplasm condensation, and dismantling of the central vacuole into several dispersed small vacuoles (Golinowski *et al.*, 1996). Concomitant with the intercellular changes, the cell walls with neighbouring cells start to dissolve at plasmodesmata. This process leads to integration of neighbouring cells into a syncytial polynuclear structure. As cell fusion continues, finally comprising several hundreds of integrated cells, the outer cell walls of the syncytium thicken in order to withstand increasing turgor pressure (Golinowski *et al.*, 1996; Grundler *et al.*, 1998). Two weeks after infection, adult males become vermiform and mobile, and leave the plant roots in search of a female mating partner. The females produce eggs and finally die, forming cysts that contain quiescent infective juveniles.

As obligate parasites, the nematodes are fully dependent on plant-derived nutrients and solutes imported into the established feeding structures. Thus syncytia have evolved as sink tissues in plant roots that are simultaneously characterized by solute and nutrient losses due to nematode feeding (Böckenhoff *et al.*, 1996). The consumption of plant-derived matter and extensive morphological re-arrangements of the affected plant cells suggest increased biosynthetic and metabolic activity linked to a high energy demand. Currently, little is known about the changes in metabolic processes during syncytium development. In recent years, several transcriptomic studies have been performed in order to improve our understanding of plant–nematode interactions (Itaya *et al.*, 1988; Puthoff *et al.*, 2003; Bar-Or *et al.*, 2005; Hammes *et al.*, 2005; Jammes *et al.*, 2005; Szakasits *et al.*, 2009). Most of these studies showed changes in the expression of genes involved in primary metabolism. These observations led to the hypothesis of a nematode-triggered remodelling of plant primary metabolism, but this lacks global experimental verification. Changes in gene expression may indicate but do not necessarily reflect the full extent of metabolic reprogramming. Initial studies have addressed parts of the metabolic systems to complement gene expression analyses (Hofmann *et al.*, 2008, 2009; Wieczorek *et al.*, 2008). In one of these studies, the starch metabolic pathway was found to be transcriptionally induced. Altered starch utilization in nematode-induced syncytia was supported by high syncytial starch accumulation (Hofmann *et al.*, 2008). One of the few metabolic studies on plant parasite interactions used a combined protein and metabolite profiling approach to study the molecular mechanisms of the *rgh1-*mediated nematode resistance response in soybean (Afzal *et al.*, 2009). Integrative analyses demonstrated effects on the relative abundances of maltose and an unknown metabolite that were associated in the two investigated plant lines with altered protein levels of glucose-6-phosphate isomerase and isoflavone reductase (Afzal *et al.*, 2009).

Metabolite profiling is a valuable and well-established method to study plant responses to abiotic and biotic stresses (Colebatch *et al.*, 2004; Kaplan *et al.*, 2004, 2007; Desbrosses *et al.*, 2005; Sanchez *et al.*, 2008; Depuydt *et al.*, 2009; Parker *et al.*, 2009). Pathogen-mediated defence responses involve physico-chemical processes, such as cell-wall modifications, and biochemical responses, such as the generation of reactive oxygen species. Such defence responses are of high energetic cost, and are based on modifications of the physiology and metabolism of infected plants both locally and systemically (Bolton, 2009). Accordingly, infection by the plant pathogenic fungus *Magnaporthe grisea* induces oxidative stress responses that are accompanied by increased energy consumption (Parker *et al.*, 2009). Other microbes, pathogens as well as symbionts, frequently induce the formation of local sink organs and increased amino acid levels (Colebatch *et al.*, 2004; Desbrosses *et al.*, 2005; Depuydt *et al.*, 2009; Parker *et al.*, 2009). Metabolic profiling approaches may also unravel the strategies that plant pathogens use to manipulate their hosts into providing and synthesizing nutrients that are essential for the pathogen. One of the best investigated plant pathogens is *Agrobacterium tumefaciens*, which induces the synthesis of opines that cannot be utilized by the plant (Beck Von Bodman *et al.*, 1992). The same strategy has been described for infection by the necrotrophic fungus *Sclerotinina slerotiorum* that triggers conversion of plant carbohydrates into fungal polyols (Jobic *et al.*, 2007).

Nematode-induced syncytia comprise small root regions that undergo enormous proliferation. The dramatic reorganization of infected plant cells as well as the high nutrient and energy demands of the pathogen suggest that severe changes occur in the plant primary metabolism. Furthermore, nematodes may be expected to trigger the specific biosynthesis of essential nutrients for their diet, and thus new metabolic pathways may be induced within the host plants. In the present study, we used a metabolic profiling approach in order to study the primary metabolism of the affected plant tissue after nematode infection. We compared syncytium samples to non-infected control roots and studied the direct local effects of nematode parasitism. Furthermore, we analysed potential systemic effects in the roots and shoots of infected plants. Gene expression analyses were used for the integrated study of potential transcriptional regulation events in syncytial metabolic processes.

## Results

Non-targeted metabolic profiling with a focus on primary and small secondary metabolites (e.g. Kaplan *et al.*, 2007) was performed by GC–MS. For a time-course analysis, root, shoot and syncytial tissues were collected at three time points that covered all juvenile stages of nematode development during plant pathogenesis. At 5 days after inoculation (dai), nematodes are in the second juvenile stage (J2), at 10 dai they are in the third developmental stage (J3) and sexually differentiating, and at 15 dai, fourth-stage juveniles (J4) become adult nematodes. Syncytia, parts of adjacent root material without infection (i-roots) and shoots of the same specimens (i-shoots) were sampled from nematode-infected plants. As controls, shoots (c-shoots) and roots (c-roots) were harvested from non-infected plants at equal developmental stages of the plant. Control plants were cultivated in parallel under otherwise equal growth conditions. The three-factorial experimental design, comprising (i) the plant organs, (ii) the presence and absence of parasitic interaction, and (iii) three critical time points after inoculation, allowed us to draw conclusions on both the developmental effects of the plant pathogenesis as well as local and systemic plant–nematode interactions.

### Independent component analysis reveals a specific metabolic pattern triggered by nematode infection

**Figure 1:**
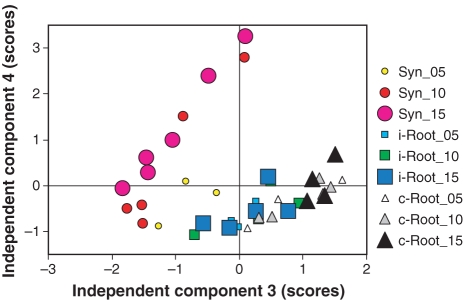
Independent component analysis (ICA) of the major metabolic variance in roots compared to syncytium samples at 5, 10 and 15 dai. Independent components that did not reflect the experimental design, namely plant-to-plant variation and technical noise, were omitted.

In order to present a global view of the obtained dataset, an independent component analysis (ICA) was performed (Figure 1). Dynamic changes of the metabolic composition in studied tissues during nematode development were investigated. The results of the analyses showed a clear distinctive metabolic profile for syncytia compared to root tissue based on the IC3 and IC4 scores (Figure 1). While the metabolic profile at the earliest stage of syncytium differentiation (5 dai) was similar to that of the non-infected roots, older syncytia (10 and 15 dai) were clearly differentiated from the initial root tissue. Further, scores for the i-root samples were scattered slightly closer to those for the syncytial samples than the scores for c-roots were (Figure 1), suggesting that the morphologically non-modified root segments of infected plants are nevertheless affected at the metabolic level by nematode parasitism. Shoot tissues of infected and non-infected plants also exhibited metabolic differentiation (Figure S1), as indicated by almost bimodal sample classification according to the scores of IC2. The metabolic trend revealed by IC3 was highly similar in i-shoots and c-shoots and was therefore attributed to plant development alone.

### Major metabolite changes during nematode development

**Figure 2:**
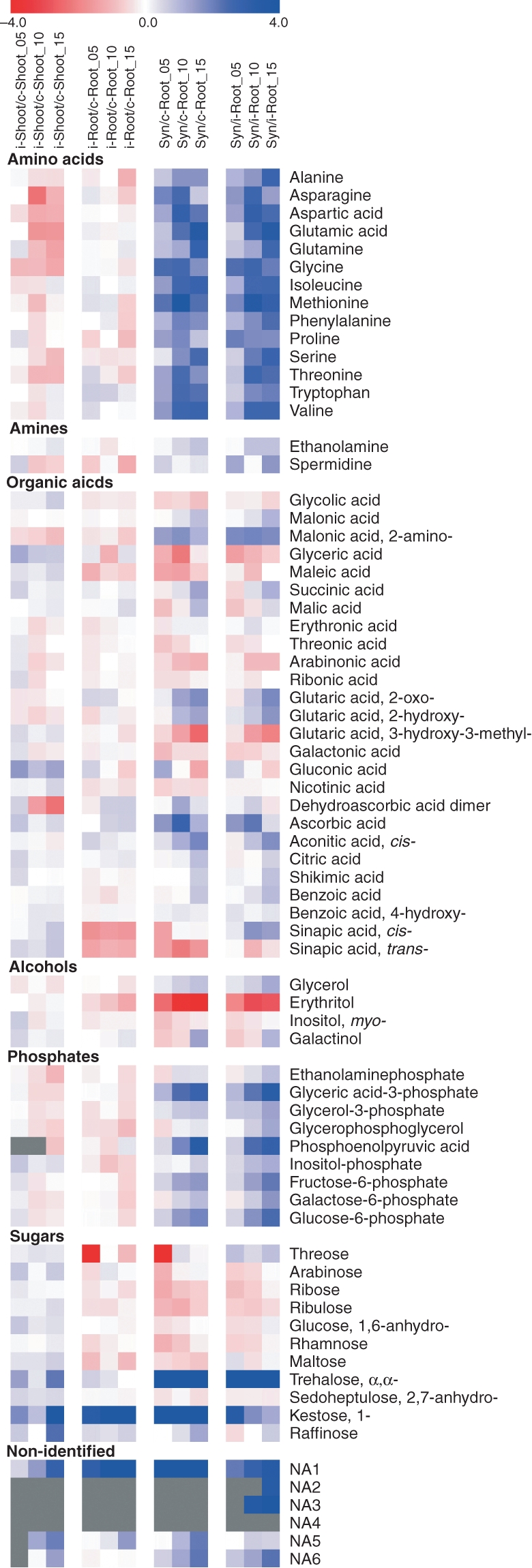
Heatmap showing changes of metabolite levels (log_2_) comparing i-shoots with c-shoots, i-roots with c-roots, syncytia with c-roots, and syncytia with i-roots. Blue indicates increased metabolite levels, and red represents decreased levels (see colour scale bar); grey indicates levels below the detection limit. Metabolites were arranged in the major biochemical classes: amino acids, amines, organic acids, alcohols, phosphates, sugars and non-identified analytes (NA1–6).

The GC–MS fingerprinting analysis by TagFinder software (Luedemann *et al.*, 2008) comprised 2142 clusters (cluster size ≥3) of correlated mass fragments that belonged to 396 time groups. Based on the reference library from the Golm metabolome database (GMD; Kopka *et al.*, 2005), 75 metabolites were identified that showed a significant response with regard to the three-factorial experimental design. In order to provide an overview of the obtained results, a heatmap was created illustrating metabolite changes at the three time points of nematode infection. In Figure 2, shoot tissues, root tissues and syncytia of infected plants are compared with roots and shoots of control plants. Fold change values (log_2_) are shown in blue if the level of the metabolite increased in the infected tissue and in red if the level of the metabolites decreased. In the heatmap display, metabolites are organized according to major biochemical groups, namely amino acids, amines, organic acids, alcohols, phosphorylated metabolites and sugars. Furthermore, the results for six non-identified metabolites are presented that were found to be highly enriched or exclusively present in syncytia or that showed strong systemic effects (see below).

Pronounced changes were observed in metabolite levels when comparing syncytia with c-roots (Figure 2). The majority of the affected metabolites increased in syncytia, such as amino acids and phosphorylated metabolites as well as certain sugars and organic acids. Only a few metabolites showed higher levels in c-roots, such as erythritol and some organic acids. Differences between syncytia and i-roots showed similar results as for the c-roots (Figure 1) but were often less pronounced. As plants were cultivated on low-sugar medium (see Experimental procedures), sucrose, glucose and fructose were omitted from this analysis.

In order to provide more detailed information, metabolites that were highly affected by nematode infection are listed in Table 1. The entire data set is presented in Table S1. In syncytia, 1-kestose and α,α′-trehalose were the most increased metabolites compared with c-roots. Furthermore, several amino acids were significantly enriched in syncytia, as were glyceric acid-3-phosphate and phosphoenolpyruvate.

**Table 1:** Metabolite pools most strongly affected by nematode infection

	Syncytia/c root			Syncytia/syncytia		
Metabolite	5 dai	10 dai	15 dai	10 dai/5 dai	15 dai/10 dai	15 dai/5 dai
1-kestose	101.08*	67.39***	37.91***	0.758	1.317	0.998
α,α-trehalose	33.40**	50.08*	21.40*	1.68	0.476**	0.8
Glutamic acid	1.76	5.08**	11.62***	2.16	2.655**	5.72***
Phosphoenolpyruvic acid	1.41	3.70	9.85*	3.97**	1.713	6.80*
Glutamine	1.82	2.40	7.52**	1.22	3.469**	4.22**
Glycine	5.44***	5.80*	7.01*	0.898	0.823	0.739**
Isoleucine	2.81**	6.02**	7.01***	1.847	1.564**	2.888***
Glyceric acid-3-phosphate	2.05	5.17**	6.94*	2.02	1.61	3.252**
Valine	2.93*	6.44*	6.66***	2.438**	1.385	3.376***
Serine	1.26	3.28	5.60**	2.92	2.447	7.15**
Methionine	4.82	14.75	5.09**	3.478	1.121	3.898
Aspartic acid	2.46	7.92	4.62**	1.804	1.283	2.315**
Galactinol	0.60	0.70	2.66*	1.27	4.369***	5.55***
Raffinose	1.00	1.34	2.47***	1.90	3.769***	7.17***
Ascorbic acid	3.01	6.97**	2.18	1.803	1.343	2.421
3-hydroxy-3-methyl glutaric acid	0.69	0.38*	0.18***	−2.28*	−2.36	−5.36**
Erythritol	−5.31***	−13.79*	−16.75*	−2.82***	−1.64	−4.64***

Values are the means of fold changes comparing the metabolite pools in syncytia to the pools in c-roots (syncytia/c-roots) at the same time point, and comparing the metabolite pools during nematode development (syncytia/syncytia). Asterisks indicate statistically significant differences (*t* test:

* *P*< 0.01,

** *P*< 0.05,

*** *P* < 0.001).

As syncytia undergo extensive and progressive re-organization during nematode development, we expected concomitant temporal changes in metabolite levels. Raffinose, serine and phosphoenolpyruvic acid were found to have the most dramatic increases over the course of nematode development (from 5 to 15 dai) (Table 1). In addition, the levels of galactinol, glutamic acid and glutamine were substantially increased in syncytia. Erythritol was the only metabolite that was strongly decreased in syncytia and that, in addition to 3-hydroxy-3-methyl-glutaric acid, exhibited a strong decrease during nematode development.

In order to elucidate any potential unprecedented markers of nematode infection, we followed certain criteria: (i) absence or levels marginally above the detection limit in c-root samples, (ii) presence in syncytia, and (iii) strong accumulation over the course of 15 days of nematode infection. As a result of this screening, four analytes (NA1–NA4) of major interest were discovered (Figure S2). The marker analytes had not been previously observed according to an exhaustive search of the Golm metabolome database (GMD). Mass spectral matching to the reference spectra of the GMD demonstrated the similarity of NA1–4 to fructose-containing trisaccharides. NA1 had the best mass spectral match with raffinose (d-Galp(1α→6)-d-Glcp(1α→2β)-d-Fruf) (Figure S2c), NA2 and NA3 matched 1-kestose (d-Glcp(1α→2β)-d-Fruf(1→2β)-d-Fruf) (Figure S2d–e), and NA4 matched melezitose (d-Glcp(1α→3)-d-Fruf(2β→1α)-d-Glcp) (Figure S2f). Matching the retention indices recorded for NA1–4 to the GMD reference data indicated that NA1, NA2 and NA4 fell into the range for trisaccharides, but were not identical with their best matching compounds. NA3 eluted well beyond the range for trisaccharides at the upper temperature limit of routine GC–MS-based profiling, which typically does not allow the detection of tetrasaccharides. In conclusion, nematode infection elicits not only the production of raffinose and 1-kestose, but also of a family of structurally related trisaccharides or similar-sized conjugates of disaccharides with as yet unknown aglycones.

### Nematode infection induces systemic changes in metabolite composition

**Figure 3:**
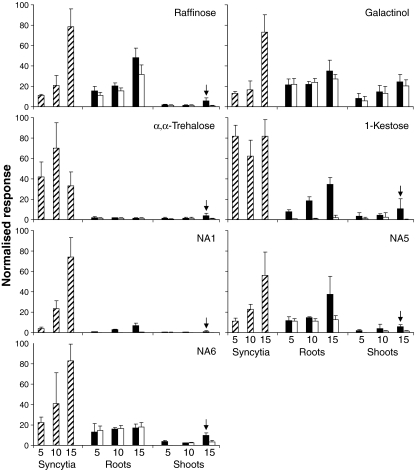
Normalized responses of raffinose, galactinol, α,α′-trehalose, 1-kestose and of the non-identified analytes NA1, NA5, NA6 in infected (black) and non-infected (white) plant tissues at 5, 10 and 15 dai. The metabolites and non-identified analytes were chosen based on their local induction in syncytial tissue and systemic responses in infected shoot tissue (i-shoots) at 15 dai (arrows). Some responses were accompanied by systemic effects in i-root tissue. Values are means ± SD.

In addition to causing local effects in the primary metabolism and oligosaccharide biosynthesis, nematode infection triggers systemic changes in metabolite levels. In i-roots only a few metabolites were changed, such as 1-kestose and *cis-* and *trans-*sinapic acid (Figure 2). In comparison with the roots, the shoots of nematode-infected plants showed a much stronger systemic response. Amino acid levels decreased compared to c-shoots, but levels of some organic acids such as glyceric acid and gluconic acid increased (Figure 2). Major systemic increases in i-shoot tissue were seen for α,α′-trehalose, 1-kestose, raffinose and its precursor galactinol levels (Figure 3). 1-kestose also showed gradual systemic accumulation over the course of nematode development (Figure 3 and Table S1). A similar pattern was observed for NA1 and two further unknown analytes (NA5 and NA6). These analytes showed a steady increase in syncytia that was coupled with a clear systemic increase in i-shoots at 15 dai (Figure 3). The presence of NA5 and NA6 clearly supports the observation of a systemic response to nematode infection; however, the chemical nature of these analytes remained elusive because co-elution of other analytes interfered with the deconvolution of high-quality mass spectra. For the purpose of analyte recognition and future elucidation, we report the characteristic unique mass fragments of NA5 and NA6 (Table S1).

### Nematodes reprogram the metabolism of infected plants

**Figure 4:**
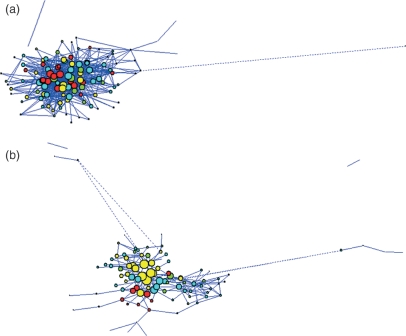
Metabolic network analysis based on Pearson’s correlation of metabolite pool size changes in (a) syncytia and (b) c-roots. Yellow nodes indicate amino acids, green nodes indicate sugars and red nodes indicate phosphates. Blue nodes indicate miscellaneous metabolites. The node size indicates the number of significant correlations of a metabolite (the so-called degree). The dark blue edges represent significant correlations (−0.7 < *r* > 0.7; *P*< 0.05). Solid lines indicate positive correlations; dotted lines indicate negative correlations.

In order to study potential nematode-triggered reprogramming of the plant metabolism, we performed metabolic network analyses based on metabolite–metabolite correlations. Correlations of the relative pool size changes within a metabolic network have been suggested to reflect the underlying enzymatic pathway system, and were shown *in silico* and experimentally to be highly dynamic when responding to environmental changes (Steuer *et al.*, 2003). Accordingly, we created comprehensive Pearson’s correlation matrices of all identified analytes separately for each of the studied tissues (Table S2). We accepted only significant correlations (*P*< 0.05) with a correlation coefficient (−0.7 < *r* > 0.7) as representing physiologically meaningful metabolite interactions. An energy-optimized layout of the resulting metabolite correlation networks, using the Kamada–Kawai algorithm (Pajek software; http://pajek.imfm.si/doku.php), was used in order to demonstrate the strongly enhanced correlation of metabolite pools in developing syncytia (Figure 4). Each node represents a metabolite pool that is colour-coded according to biochemical classes. The node size indicates the number of significant correlations (the node degree). Blue lines (edges) represent significant correlations (*P* < 0.05; *r* > 0.7) between the nodes; dashed lines indicate negative correlations (*P*< 0.05; *r* < −0.7). The location of the node indicates its degree of connectedness, such that a more central position indicates a central role in the metabolism reprogramming. In comparison with c-roots, the metabolic network of syncytia show a profoundly enhanced density, a higher number of metabolites interlinked by significant correlations, and a higher number of highly connected metabolites, so-called hubs (Figure 4a). Among the metabolite classes that constitute the networks, a clear shift becomes apparent. In c-roots, amino acids are the most frequently connected constituents of the correlation network (Figure 4b). In syncytial tissue, the proportion and connectivity of phosphorylated metabolites are highly increased. Systemic effects on the metabolic correlation networks are present but less pronounced (Figure S3). Shoot tissues of infected plants are characterized by a higher number of nodes and significant correlations. Phosphorylated metabolites appear to play a more central role in i-shoot correlation networks, as observed in the root tissues. In addition to the differentially accumulating metabolites (see above), this network property indicates a systemic plant response to nematode infection.

In order to reveal the changes among the metabolic hubs of the networks, we analysed the number of significant correlations of each metabolite separately in syncytial, c-root, i-shoot and c-shoot networks, respectively (Table S3). The most connected metabolites in syncytial networks were inositol phosphate, ribulose and threonic acid. In contrast, the hubs in c-roots were methionine and asparagine, with a hub of ribonic acid in i-roots. The hubs in i-shoots were ribonic acid and glycerol-3-phosphate, and *myo-*inositol was the most connected metabolite in c-shoot networks (Table S3).

Further, changes in metabolites connectedness in terms of the number of significant correlations when comparing syncytium with c-root, i-shoot and c-shoot networks were analysed (Figure S4). In the syncytial network (Figure S4a), *m*yo-inositol phosphate, ethanolamine phosphate and ribulose had 35–40 more connections than the c-root network. Moreover, benzoic acid had 27 correlations in syncytia but none in c-roots. In total, 51 more metabolites showed correlations in the syncytial network compared to c-roots. In the control, only 10 metabolites had more correlation than in syncytia, the top-ranking nodes were the amino acids proline, glycine and asparagine (Figure S4a). Interestingly, the increases in 1-kestose and α,α′-trehalose, which are the highest increases in nematode-induced syncytia compared to non-infected control roots (Table 1), appear to be isolated responses, as they hardly correlate with other metabolites. The systemic differences in metabolite correlation frequencies were much smaller than in directly affected tissues (Figure S4b). A set of 30 metabolites correlated more often in the i-shoot network, and 22 were more connected in c-shoots.

### Integration of system levels reveals potential transcriptional regulation of specific metabolic processes

**Figure 5:**
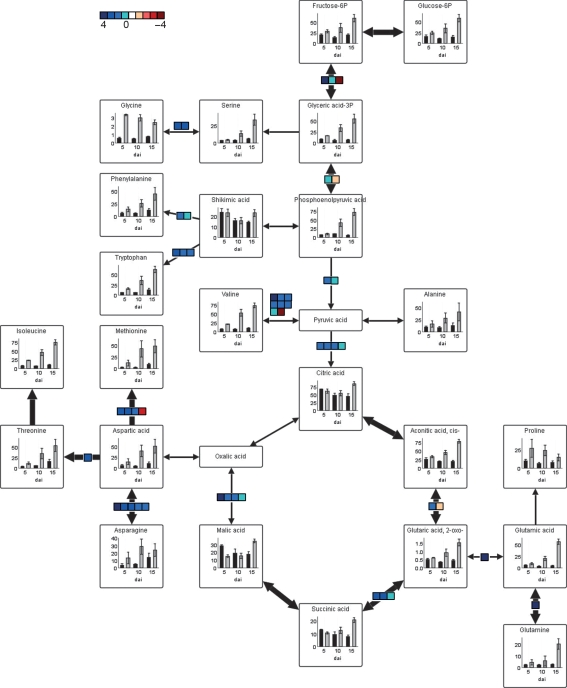
Integrated analysis of the metabolic profiles (bar diagrams) and gene expression profiles (colour codes) of the glycolysis and TCA cycle pathways. The time series for c-root development (black) is compared to that for syncytial development (grey). Bar charts show normalized metabolite responses (means ± SE) at 5, 10 and 15 dai. Bold arrows indicate significant correlations of metabolites (Pearson’s correlation analysis, *P*< 0.05, correlation coefficient −0.7 < *r* > 0.7). Colour coding indicates the fold changes (log_2_) of significantly affected genes in the various gene families according to previously published GeneChip results (Szakasits *et al.*, 2009). Blue indicates significant up-regulation and red indicates significant down-regulation.

Through the current study, data have become available to allow integration of metabolic phenotypes and transcriptomic phenotypes (Szakasits *et al.*, 2009) to characterize the nematode-induced development of plant root tissue into a syncytial feeding structure. Potential transcriptional regulation mechanisms for metabolic reactions were investigated by comparing the two datasets. A metabolic pathway scaffold of glycolysis, the tricarboxylic acid (TCA) cycle and connected branch points towards amino acid biosynthesis was generated (Figure 5). The relative changes in metabolite pools (Figure 5; bar diagrams) and enzyme gene expression (Figure 5; heatmaps of significantly affected genes of the relevant gene families) show integrated activation of the primary metabolism in syncytia compared with c-roots. The high enrichment of most amino acids as well as phosphorylated intermediates of glycolysis is matched by significantly increased expression levels of pathway-linked genes. Only a few of the genes encoding the enzymes involved in glycolysis were significantly down-regulated in syncytia. Most organic acids of the TCA cycle showed few differences between syncytia and c-roots at 5 and 10 dai, but their levels started to increase in syncytia at the later stage of nematode development (15 dai). The concomitant strong up-regulation of TCA cycle-related genes suggests a high demand for intermediates of this pathway.

In order to compare transcriptome profile data obtained in an earlier study (Szakasits *et al.*, 2009) with the experimental setup of the metabolic experimentation in the present study, representative c-root and syncytium samples were collected under growth and sample handling conditions that were identical to those used for the current metabolic profiling analysis (see Experimental procedures). The generated RNA samples were subjected to quantitative RT-PCR to analyse the expression of selected genes that encode enzymes of amino acid biosynthesis, glycolysis, the TCA cycle and sugar metabolism (Table 2). All selected genes were significantly up-regulated in syncytia and showed a temporal pattern over syncytial development. Expression values obtained by quantitative RT-PCR generally confirmed the GeneChip data but also showed some deviations. Specifically, the two genes *GAPA2* (At1g12900) and At3g23940 showed stronger nematode induction at 15 dai compared with the results of the GeneChip analyses, and two other genes, *MTO2* (At4g29840) and *TSA1* (At4g02610), were induced to a lower extent as judged by quantitative RT-PCR (Table 2).

**Table 2:** Fold changes in expression (FC) (log_2_) of genes encoding enzymes involved in the primary metabolism of nematode-induced syncytia were studied by quantitative RT-PCR at 5, 10 and 15 dai and compared with GeneChip data (5 + 15 dai)

	5 dai			10 dai		15 dai		GeneChip data*			
Locus	EC	FC	SE	FC	SE	FC	SE	FC	Gene name	Description	Metabolism
At1g12900	1.2.1.9	1.83	0.46	6.30	2.26	11.52	6.24	4.6	*GAPA-2*	Glyceraldehyde 3-phosphate dehydrogenase A, subunit 2	Glyc
At3g23940	4.2.1.9	4.22	0.35	7.94	2.19	7.59	2.14	4.0	*AADH*	Dihydroxy acid dehydratase	AA
At2g30970	2.6.1.1	2.42	0.18	3.53	0.17	2.91	0.45	4.3	*ASP1*	Aspartate amino transferase 1	AA
At4g32520	2.1.2.1	1.68	0.40	2.29	0.72	2.81	0.71	3.5	*SHM3*	Serine hydroxymethyl transferase 3	AA
At1g35910	3.1.3.12	3.28	0.84	2.89	1.61	2.68	1.72	2.8	*TPPd*	Trehalose phosphate phosphatase	S
At3g54090	2.7.1.4	2.08	0.13	3.65	0.29	2.59	0.10	2.4	*FK*	Fructokinase	S
At3g63250	2.1.1.14	2.08	0.72	2.06	0.75	2.57	0.33	2.3	*HMT2*	Homocysteine methyltransferase 2	AA
At1g69370	5.4.99.5	1.94	0.57	1.85	0.20	2.20	0.63	2.7	*CM3*	Chorismate mutase	AA
At5g14590	1.1.1.42	1.87	0.17	2.44	0.41	2.09	0.30	2.2	*IDH*	Isocitrate dehydrogenase	TCA
At4g29840	4.2.3.1	1.31	0.48	1.51	0.33	1.67	0.19	3.1	*TS/MTO2*	Threonine synthase	AA
At4g02610	4.2.1.20	1.34	0.24	1.36	0.18	1.58	0.23	3.3	*TSA1*	Tryptophan synthase, α subunit	AA

* The Gene Chip data have been published previously (Szakasits *et al.*, 2009). Values are means ± SE (*n* = 3).

AA, amino acid metabolism; Glyc, glycolysis; S, sugar metabolism; TCA, tricarboxylic acid cycle.

Potential transcriptional regulation of metabolic processes was studied by Pearson’s correlation analysis. Gene versus gene (G:G) and metabolite versus metabolite (M:M) fold change levels were correlated. Transcriptional changes of several genes encoding enzymes involved in amino acid biosynthesis showed significant correlations (G:G) (Table 3). In contrast, the expression levels of *ASP1* (At2g30970) and *TPPd* (At1g35910) did not significantly correlate with those of any other transcript. For the M:M correlations, metabolites that were substrates or products of the enzymes encoded by the studied genes (focus metabolites) were used. Almost all metabolite fold changes showed significant and positive correlations (Table 3), indicating high coordination of the syncytial primary metabolism. α,α-trehalose was the only metabolite that did not correlate significantly with any of the other focus metabolites. A comparison of the results of the two Pearson’s correlation analyses revealed close affinities between some M:M and G:G correlation coefficients of the same metabolic pathway. Accordingly, the fold change levels of tryptophan and phenylalanine correlate significantly, as do the fold changes in gene expression of the relevant genes *CM3* and *TSA1*.

**Table 3:** Pearson's correlation analysis of the relative changes in metabolite pool sizes and corresponding transcript abundances in nematode-induced syncytia

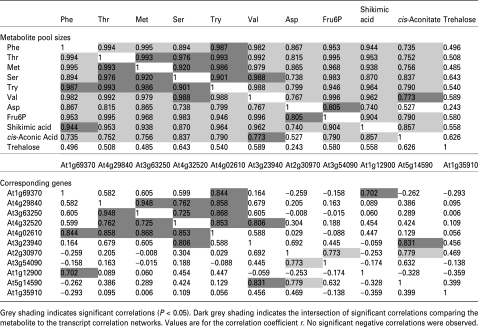

## Discussion

Pathogen-triggered plant defence responses and the exploitation of infected plant tissues are energy- and nutrient-intensive for the hosts (Bolton, 2009). In addition to local manipulations, pathogen attack induces systemic responses, leading to reduced biomass production or crop yield losses. Nematode juveniles suppress the expression of plant defence-related genes shortly after induction of feeding structures (Szakasits *et al.*, 2009) and transform the affected plant cells into syncytia. In order to study the metabolic changes triggered by nematode infection, a metabolic profiling approach was chosen. Formed by incorporating cells in the closest vicinity, syncytia are locally highly restricted and become a strongly modified and proliferated cell complex. Therefore, small but highly affected root sections can be sampled, and juveniles of the beet cyst nematode can be removed easily to obtain pure plant materials.

### Nematodes reprogram the biosynthesis of plant primary metabolites

Of the various metabolite groups, amino acid biosynthesis was most globally affected by nematode infection, a pattern frequently observed in plant–microbe interactions (Colebatch *et al.*, 2004; Desbrosses *et al.*, 2005; Depuydt *et al.*, 2009; Parker *et al.*, 2009). Plant pathogens are net consumers, and fully depend on organic nitrogen and essential amino acids from their hosts. Amino acids are vital for protein synthesis and are precursors for a large number of metabolites. In plants, sink tissues are supplied with organic nitrogen forms from source tissues (Roessner *et al.*, 2001; Jeong *et al.*, 2004). In syncytia, the levels of the major transport amino acids glutamic acid, aspartic acid and glutamine increased significantly compared to c-roots. Aspartic acid, one of the central regulators for carbon/nitrogen metabolism, appears to play a specific role in syncytia. Many aspartic acid-derived amino acids were enriched in syncytia and correlated closely with the levels of aspartic acid. Induction of these pathways was supported by significantly increased gene expression of the involved enzymes. Accordingly, the present study showed that methionine was highly enriched in syncytia, which matched previous results showing up-regulation of methionine-related transcripts (Szakasits *et al.*, 2009). Methionine is an essential amino acid and an important sulfur source in animal diets. Furthermore, the amino acid is a signal for the initiation of mRNA translation. In the Yang cycle, methionine is involved in ethylene production as a precursor of 1-aminocyclo-propane-1-carboxylate (ACC), which has been shown to promote nematode infection and development (Goverse *et al.*, 2000; Wubben *et al.*, 2001). Further, the shikimic acid-based aromatic amino acids tryptophan and phenylalanine were highly enriched in syncytia. The unchanged levels of shikimic acid and the simultaneous up-regulation of genes encoding aromatic amino acid biosynthesis-related enzymes indicate high utilization of this pathway. The shikimic acid-dependent pathways may also be induced by nematode-secreted chorismate mutases (Lambert *et al.*, 1999; Jones *et al.*, 2003). In syncytia, tryptophan may be processed into secondary metabolite biosynthesis. The level of tryptophan-derived benzoic acid correlated with 27 metabolites in syncytia but did not correlate with any metabolites in c-roots, indicating that it belongs to a highly activated pathway. Valine was one of the most enriched amino acids in syncytia, and the gene At3g23940 that encodes the dihydroxy acid dehydratase involved in valine biosynthesis was the second highest up-regulated gene in the current analysis. Valine is an essential amino acid and is a precursor for secondary metabolites such as glucosinolates.

In addition to amino acids, special attention was paid to a number of unusual carbohydrates. 1-kestose was the highest accumulated metabolite in all studied parts of infected plants. What is most exceptional about 1-kestose enrichment after nematode infection is that Arabidopsis is not a natural fructan accumulator such as Asteraceae, Campanulaceae or Poaceae species, in which kestose is a precursor for fructans and inulin (Cairns, 2003). Fructans are generally synthesized directly from sucrose without the involvement of phosphorylated sugars and are water-soluble. In Arabidopsis, 1-kestose may be synthesized and hydrolysed by non-specific binding of cell-wall invertase (Le Roy *et al.*, 2007) or by two putative fructan exohydrolases (de Coninck *et al.*, 2005). At present, we can only speculate about the role of 1-kestose in nematode-induced syncytia as it showed no significant correlations, providing little information about its metabolic pathway. The fructan may serve as a form of carbohydrate storage in addition to starch that was studied previously (Hofmann *et al.*, 2008). 1-kestose may also play a role in stress tolerance against cold and drought, and has been suggested to be involved in plant defence against pathogenic bacteria (Van den Ende *et al.*, 2004). Finally, 1-kestose may play an essential role in the nematode diet as a carbohydrate source. Currently, there are no published studies on the occurrence of kestose in nematodes, so it appears unlikely that 1-kestose is a nematode-derived metabolite.

α,α′-trehalose levels were also highly increased in syncytia, confirming previous results (Afzal *et al.*, 2009; Hofmann *et al.*, 2009). Trehalose plays an important role in the nematode diet, especially during reduced water availability and dormancy. Compared to other analysed sugars in syncytia, levels of the plant-specific α,α′-trehalose were low, so its role as possible carbohydrate source for feeding nematodes may be ruled out. In plants, trehalose has been suggested to act as a sugar signal, and usually accumulates at low concentrations (Schluepmann *et al.*, 2003, 2004; Lunn *et al.*, 2006; Elling *et al.*, 2007; Ramon and Rolland, 2007). Plant pathogens may use trehalose to interfere with the plant sugar signalling system, which could transform infection sites into local sink tissues (Brodmann *et al.*, 2002). The role of trehalose as sugar signal during nematode development may explain the strong systemic increases and moderately affected gene expression. Several genes encoding trehalose phosphate synthase are down-regulated, and *TPPd* was the only significant up-regulated gene encoding trehalose phosphate phosphatase (Szakasits *et al.*, 2009). However, the up-regulation of *TPPd* did not reflect the highly increased α,α′-trehalose levels in syncytia.

Although carbohydrate depletion has been reported in other plant–pathogen interactions (Colebatch *et al.*, 2004; Desbrosses *et al.*, 2005), syncytia are characterized by increased sugar levels. In addition to the present study, previous studies have reported elevated soluble sugar pools and starch levels in syncytia (Hofmann *et al.*, 2007, 2008, 2009). The present finding of a high number of significant correlations of *myo*-inositol phosphate with other metabolites in syncytia support the previously described importance of the *myo*-inositol oxygenase pathway in nematode-induced syncytia (Siddique *et al.*, 2009).

### Nematodes trigger the formation of a particular metabolic niche in plant roots

Nematode-induced syncytia have previously been suggested as a newly established metabolically highly active sink tissue in plant roots (Böckenhoff *et al.*, 1996; Golinowski *et al.*, 1996; Szakasits *et al.*, 2009). In the present study, high induction of the primary metabolism in syncytia was reflected by increased levels of many metabolites. Further, the high complexity of the correlation-based network analysis in syncytia indicates activation and coordination of the underlying enzymatic reactions. Thus, metabolites that significantly correlate with a large number of other metabolites, such as *myo*-inositol phosphate, play a central role in syncytium metabolism. Furthermore, nematode-induced changes in metabolic coordination may be identified by comparing correlation analyses of syncytia with those of c-roots. For example, threonic acid is a product of ascorbic acid degradation and could indicate an oxidative stress response (Loewus, 1999). Ethanolamine phosphate is a precursor for glycerophospholipid metabolism (Keogh *et al.*, 2009), and ribulose is produced via the pentose phosphate pathway (Dennis and Blakeley, 2000).

Syncytia not only need to provide nutrients for the feeding nematodes, but incoming solutes are also required for plant cell anabolism and catabolism to cover the increasing energy consumption resulting from the induced morphological rearrangements. In order to fuel energy generation, phloem-derived sugars flow into glycolysis and consequently into the TCA cycle (Dennis and Blakeley, 2000). In the present study, we found that the levels of most of the analysed metabolites of glycolysis increase steadily during nematode development. A potential high flux through glycolysis is suggested by the significant up-regulation of most of the glycolysis pathway-related genes such as At1g12900. At1g12900 codes for the glyceraldehyde-3-phosphate dehydrogenase, which was the strongest up-regulated gene in the present analysis. Most organic acids in the subsequent TCA cycle showed low levels in early-stage syncytia but significantly increased over time. The TCA cycle is responsible for the major part of carbohydrate, fatty acid and amino acid oxidation, and produces energy and reducing power (Fernie 2004). However, activation of the TCA cycle may not be evident through increased levels of the involved organic acids due to rapid processing by subsequent reactions (Colebatch *et al.*, 2004; Rizhsky *et al.*, 2004; Desbrosses *et al.*, 2005; Baxter *et al.*, 2007; Afzal *et al.*, 2009). In syncytia, the up-regulation of genes coding for enzymes of the TCA cycle, such as At5g14590 encoding isocitrate dehydrogenase, may indicate a compensatory response to the requirement for a high metabolic flux through this pathway.

In addition to organic acids, a simultaneous increase in amino acid and sugar levels was found in syncytia. Such responses are more usually attributed to cold acclimation and drought stress (Rizhsky *et al.*, 2004; Kaplan *et al.*, 2007; Usadel *et al.*, 2008) rather than the plant–pathogen response. Thus, syncytia may suffer from osmotic stress caused by water loss through the feeding nematode, which may take up four times the volume of the syncytium contents each day (Sijmons *et al.*, 1991). Induction of the osmotic stress response was supported by the increased levels of galactinol, raffinose, α,α′-trehalose and possibly also 1-kestose. However, the level of proline one of the major metabolites that accumulates during water deficit and salt stress in plants (Kaplan *et al.*, 2004, 2007; Sanchez *et al.*, 2008) did not increase strongly in syncytia (Rizhsky *et al.*, 2004). Thus, organic nitrogen forms may be particularly important in other metabolic pathways rather than being used as compatible solutes. In syncytia, water balancing must be tightly regulated as these cells lack a central vacuole (Golinowski *et al.*, 1996). A homologous situation can be found in developing seeds, which also lack a central vacuole and demonstrate well-functioning osmo-regulation. During the desiccation period of Arabidopsis seeds, the levels of free amino acids, some organic acids and sugars such as raffinose and α,α′-trehalose increased, but only a moderate increase in proline levels was found (Fait *et al.*, 2006).

### Systemic effects of nematode infection

In addition to local manipulations, pathogen attack often affects distant plant tissues. To date, most studies of such systemic effects have focused on phytohormone status, systemic required resistance and related topics (Durrant and Dong, 2004). However, pathogen-induced sink structures may lead to depletion of certain metabolites in remote source tissues, thus inducing source leaf primary metabolism. There are only a few studies on systemic changes of metabolite levels in plants interacting with other organisms. Spider mite and caterpillar feeding on cotton leaves did not result in significant systemic changes in sugar and amino acid levels (Schmidt *et al.*, 2009). Priming of various plant species with beneficial root bacteria has been shown to systemically affect metabolism-related gene expression in plant shoots (Wang *et al.*, 2005; Cartieaux *et al.*, 2008; Sarosh *et al.*, 2009).

In the present study, clear systemic responses to nematode parasitism were observed by changes in metabolite levels and correlation-based network analyses. The systemic reduction of the major transport amino acids asparagine, aspartic acid, glutamine and glutamic acid, and their simultaneous enrichment in syncytia, underlines the strong sink character of syncytia and their high demand for organic nitrogen. Further, the systemic decrease and local increase of glycine may indicate the demand for glycine-rich proteins in syncytia (Potenza *et al.*, 2001; Karimi *et al.*, 2002). Systemically reduced levels of dehydroascobic acid oxidized from ascorbic acid may indicate oxidative stress or stimulated plant defence (de Gara *et al.*, 2003). The accumulation of 1-kestose and raffinose in i-shoots may indicate an osmotic stress response, and the systemic enrichment of α,α′-trehalose underlines its putative role in biochemical sugar signalling.

In addition to previously described metabolites, we found six non-identified analytes that showed clear systemic enrichment. These metabolites may be synthesized in the source leaves in response to a presently unknown signal, in order to be transported into root syncytia. In addition, their accumulation may be traced back to a systemic allocation from syncytia through the root phloem into the shoots. This systemic accumulation may reflect a secondary abiotic stress response induced by nematode parasitism. As the identity of those substances is not yet known, we cannot speculate about their putative role in the metabolism of infected plants.

In summary, we present a detailed metabolic profiling analysis of nematode-induced syncytia. The results showed transcriptional regulation of the highly induced primary metabolism. Further, nematodes dramatically manipulated the plants to induce a niche specialized for nematode parasitism. This includes synthesis of metabolites that do not naturally accumulate in Arabidopsis, such as 1-kestose. Such results clearly underline the role of metabolite analyses in revealing processes that occur during plant–nematode interaction but may not be detectable by transcriptome analyses. Finally, clear systemic effects of nematode parasitism could be identified. The identity of the unknown analytes should be studied, and may provide new insights into plant–nematode interactions.

## Experimental procedures

### Plant growth conditions and nematode culture

*Arabidopsis thaliana* wild-type (Col-0) seeds were germinated under sterile conditions and grown under a 16 h/8 h light/dark photoperiod (150 μmol m^−2^ sec^−1^) at 25°C. As substrate, a low-sucrose Knop medium (5 g L^−1^ sucrose) was used instead of the standard Knop medium (20 g L^−1^ sucrose). Twelve-day-old plants were inoculated with 50 freshly hatched mobile second-stage juveniles (J2) (Sijmons *et al.*, 1991) of *H. schachtii* obtained from sterile stock culture.

### Sample collection

Sampling was performed at 5, 10 and 15 days after inoculation (dai). Syncytia, non-infected root fragments adjacent to the syncytia (i-roots) and shoots of the same specimen (i-shoots) were cut. In order to eliminate wound responses of the host plants, the collected material was immediately shock-frozen in liquid nitrogen (sampling time 20–25 sec). Shoots (c-shoots) and roots (c-roots) of non-infected plants were sampled as control materials. Sampling always took place in the middle of the illumination period in order to rule out diurnal effects. Samples were collected for 3–6 independent biological replicates. To obtain 40 mg fresh weight material, 200–300 15-day-old syncytia were collected.

### GC–MS analysis

Soluble metabolites were extracted by methanol/chloroform extraction of deep frozen powder of pooled samples amounting to approximately 50 mg fresh weight (Kaplan *et al.*, 2004, 2007). A polar metabolite fraction was generated by liquid:liquid partitioning, and dried under vacuum for storage at −80°C until further processing. Samples were transported in N_2_ atmosphere on dry ice.

Chemical derivatization, namely sequential methoxyamination and trimethylsilylation, of dried extracts was performed essentially as described previously (Kaplan *et al.*, 2004; Desbrosses *et al.*, 2005). Gas chromatography coupled to electron impact ionization/time-of-flight mass spectrometry (GC/EI-TOF-MS) was performed using an Agilent 6890N24 gas chromatograph with splitless injection (http://www.agilent.com) connected to a Pegasus III time-of-flight mass spectrometer (LECO Instrumente GmbH). Metabolites were quantified using at least three specific mass fragments. Ascorbic acid was internally standardized by standard addition of isoascorbic acid. The effects of laboratory and reagent contaminations were baseline-corrected using control experiments from which the sample was omitted. Gas chromatography data pre-processing and compound identification were performed using TagFinder software (Luedemann *et al.*, 2008) and the mass spectral and retention time index collection of the Golm metabolome database (GMD; Kopka *et al.*, 2005). Thresholds for metabolite identification were a mass spectral matching factor >650 and a retention index deviation <3.0 (Strehmel *et al.*, 2008). The mass spectra of as yet unidentified metabolites were deconvoluted, manually curated (Wagner *et al.*, 2003), and uploaded into the GMD. Metabolite identifications, matching criteria and normalized responses are summarized in Table S1.

### Statistical analyses and data evaluation

Normalized responses were used for statistical analyses. Means ± standard deviation (SD), with *t* tests and anova, were used to determine relevant metabolites from the GC–MS fingerprinting data. Simple statistical operations and heatmap visualization of log_2_-transformed fold changes were performed using the software MultiExperiment Viewer version 4.4.0 (http://www.tm4.org/mev/). The independent component analysis (ICA) was performed using MetaGeneAnalyse software version 1.7.1 (http://metagenealyse.mpimp-golm.mpg.de/) (Scholz *et al.*, 2005). Pearson’s correlation analyses were performed using spss 15.0 (http://www.spss.com). The data were transformed into a Pajek import file using excel2pajek software (http://vlado.fmf.uni-lj.si/pub/networks/pajek/). Pajek 1.24 was then used to create free Kamada–Kawai metabolic networks. In order to present an integrated picture of metabolite and transcript level, vanted version 1.66 was used (Junker *et al.*, 2006).

### RNA extraction and cDNA preparation

RNA was isolated using an RNeasy plant mini kit (Qiagen, http://www.qiagen.com/) according to the manufacturer’s protocol, including DNase I (Qiagen) digestion. The quality and quantity of obtained RNA was checked using an Agilent 2100 bioanalyzer. Reverse transcription was performed using SuperScript III reverse transcriptase (Invitrogen, http://www.invitrogen.com/) and random primers [oligo(dN)_6_] according to the manufacturer’s instructions.

### Quantitative RT-PCR

Quantitative RT-PCR was performed using an abi prism 7300 sequence detector (Applied BioSystems, http://www.appliedbiosystems.com/). Each quantitative PCR sample contained 12.5 μl Platinum SYBR Green qPCR SuperMix with uracil-DNA glycosylase (UDG) and ROX Reference Dye (Invitrogen) and 2 μl cDNA, with addition of distilled water to obtain a 25 μl total reaction volume. MgCl_2_ and forward and reverse primers (10 mm, Table S4) were added in order to achieve optimal PCR efficiencies. All samples were tested in triplicate; water was used as a control to rule out false-positive signals. In addition, dissociation runs were performed to control the possible formation of primer dimers. *18S rRNA* and *UBP22*, which are known to be stably expressed in syncytia (Hofmann and Grundler, 2007), were used as internal references. Samples were diluted 1:3 and 1:100 for *18S rRNA*. Results were analysed using the sequence detection software SDS version 2.0 (Applied BioSystems).

## SUPPORTING INFORMATION

**Figure S1.** Independent component analysis (ICA) of the major metabolic variance in shoot samples at 5, 10, and 15 dai.

**Figure S2.** GC-MS mass spectra and retention time indices that allow the identification of raffinose (a), 1-kestose (b), and the classification of non-identified analytes (C-F) NA1-NA4.

**Figure S3.** Pearson's correlation-based metabolic network analysis of c-shoots and I-shoots

**Figure S4.** Difference of the number of significant correlations of single metabolites for the local and systemic responses.

**Table S1.** Entire data set of the metabolite profiling in c-shoots, i-shoots, c-roots, i-roots and syncytia.

**Table S2.** Pearson's correlation analyses.

**Table S3.** Number of correlations of metabolites in syncytia, c-roots, i-shoots and c-shoots.

**Table S4.** Primers used for quantitative RT-PCR.

As a service to our authors and readers, this journal provides supporting information supplied by the authors. Such materials are peer-reviewed and may be re-organized for online delivery, but are not copy-edited or typeset. Technical support issues arising from supporting information (other than missing files) should be addressed to the authors.
