# Supplementary material for: Metabolic profiling reveals local and systemic responses of host plants to nematode parasitism
Source: Plant J. 2010 May 11;62(6):1058–71. doi: 10.1111/j.1365-313X.2010.04217.x (PMC2904900; doi:10.1111/j.1365-313X.2010.04217.x)

Figure S1. Independent component analysis (ICA) of the major metabolic variance in shoot samples at 5, 10, and 15 dai. Independent components which did not reflect the experimental design, namely plant to plant variation and technical noise were omitted.

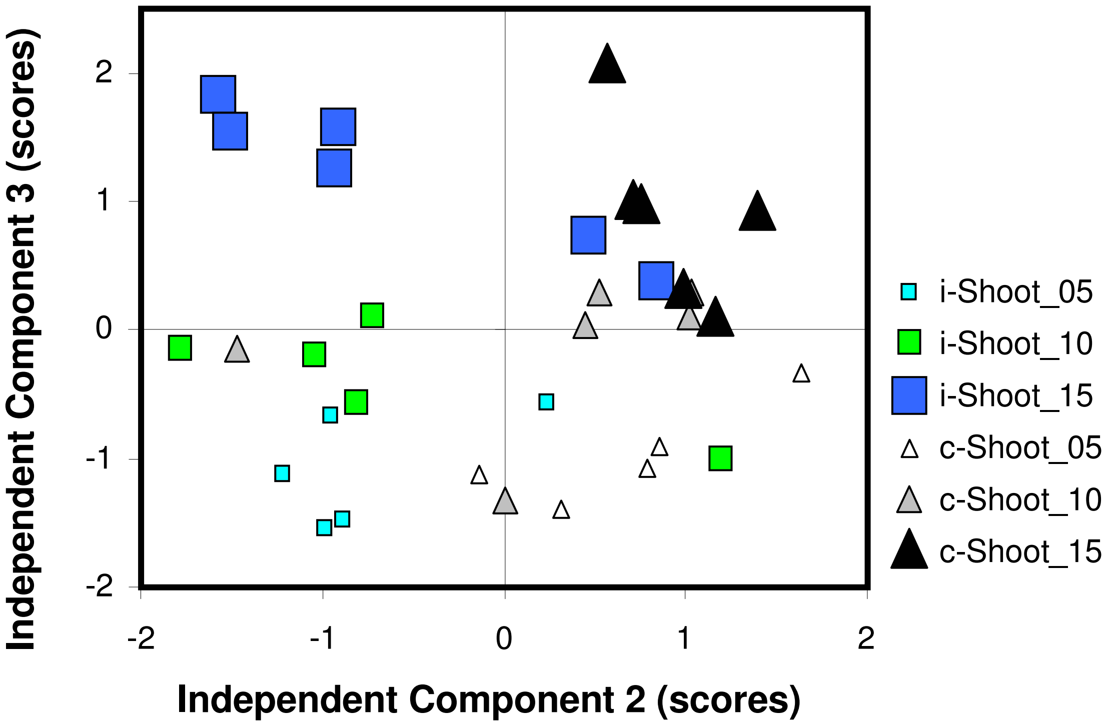

Supplement: Supplementary file 1 [file tpj0062-1058-SD1.pdf]
