# Supplementary material for: Metabolic profiling reveals local and systemic responses of host plants to nematode parasitism
Source: Plant J. 2010 May 11;62(6):1058–71. doi: 10.1111/j.1365-313X.2010.04217.x (PMC2904900; doi:10.1111/j.1365-313X.2010.04217.x)

Figure S2. GC-MS mass spectra and retention time indices that allow the identification of raffinose (a), 1-kestose (b), and the classification of non-identified analytes (C-F) NA1-NA4. The inserts demonstrate the discovered indicator properties of the chosen metabolites for the process of syncytium induction (5-15 dai) compared to non-infected c-roots at 5 dai, respectively. The identification criteria, mass spectral matching factor (MF) and retention index deviation (DRI) indicate the thresholds for a positive identification (DRI<3.0 and MF>650) compared to an analyte classification by best mass spectral match, NA1-NA4 (DRI>3.0 and MF>800).

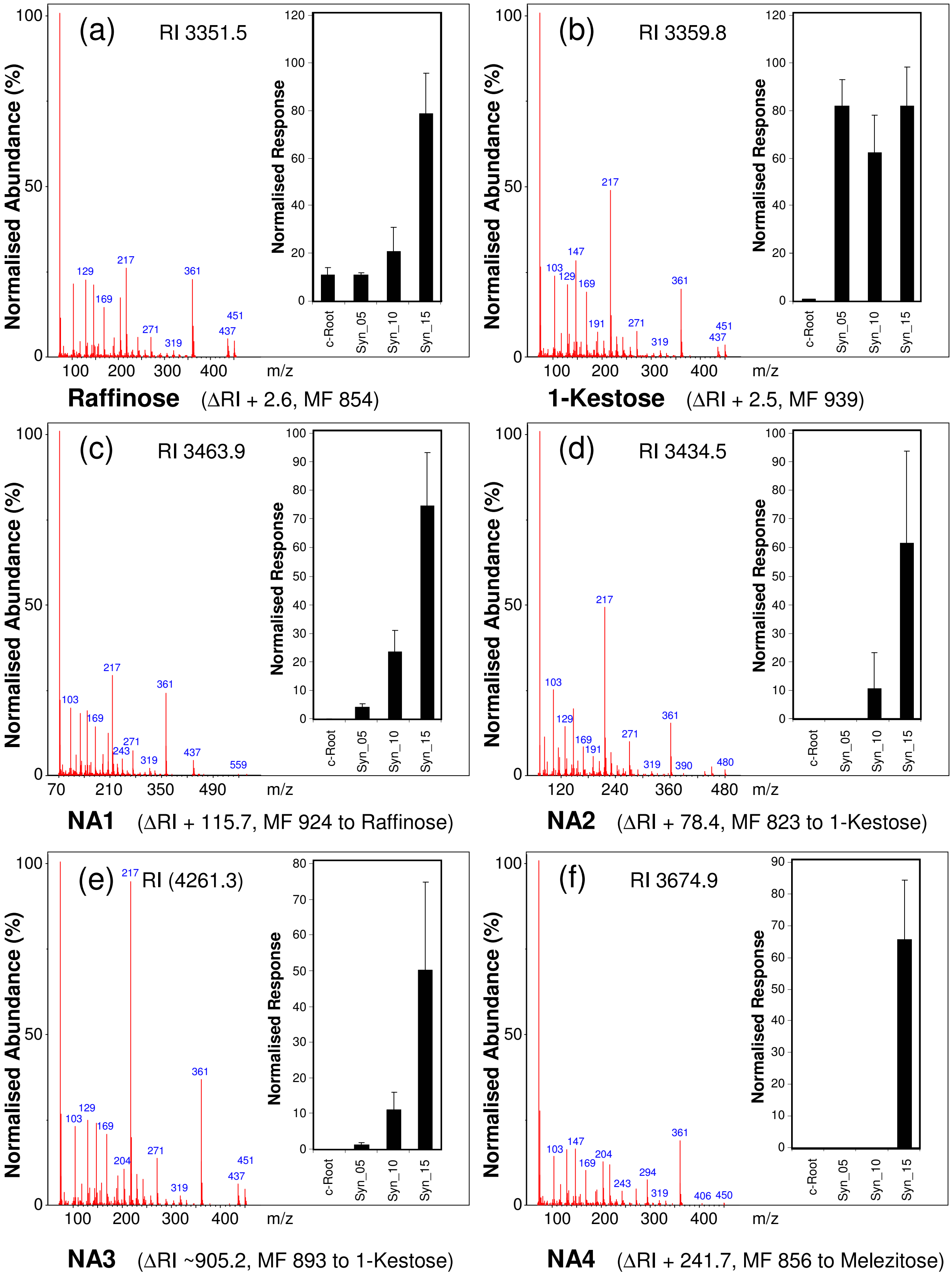

Supplement: Supplementary file 2 [file tpj0062-1058-SD2.pdf]
