# Supplementary material for: Metabolic profiling reveals local and systemic responses of host plants to nematode parasitism
Source: Plant J. 2010 May 11;62(6):1058–71. doi: 10.1111/j.1365-313X.2010.04217.x (PMC2904900; doi:10.1111/j.1365-313X.2010.04217.x)

Figure S3. Pearson's correlation based metabolic network analysis of (A) c-shoots and (B) i-shoots, according to Figure 5.

(a)

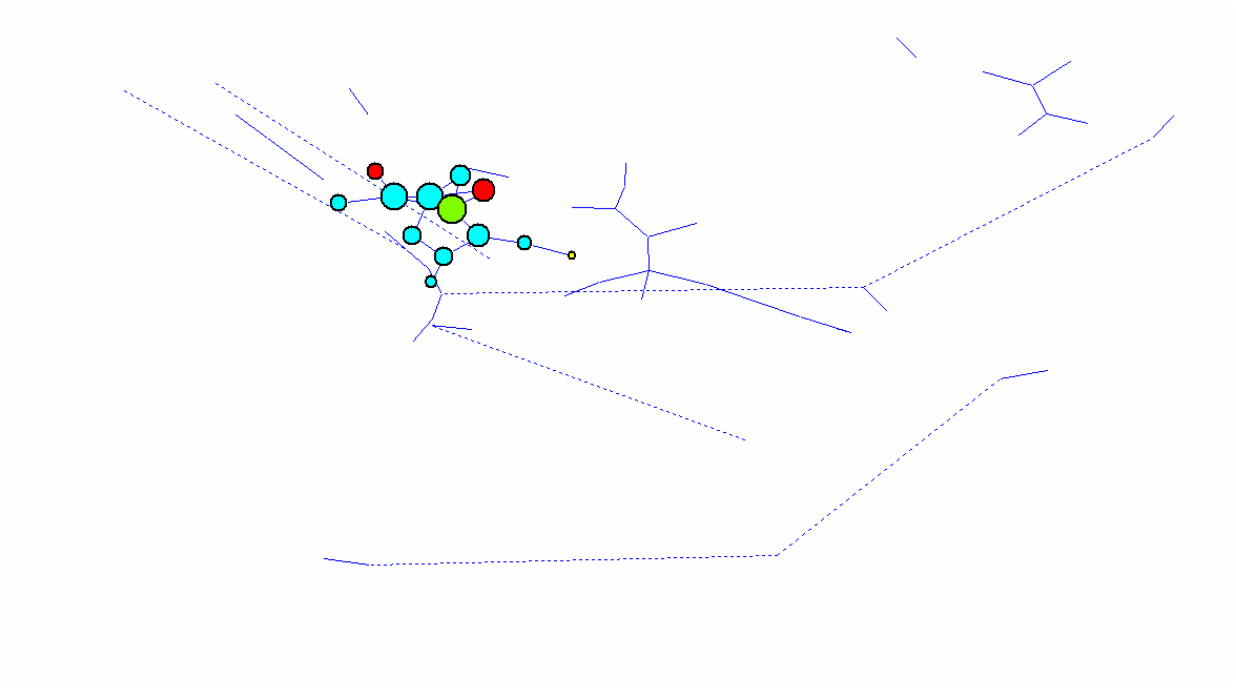

(b)

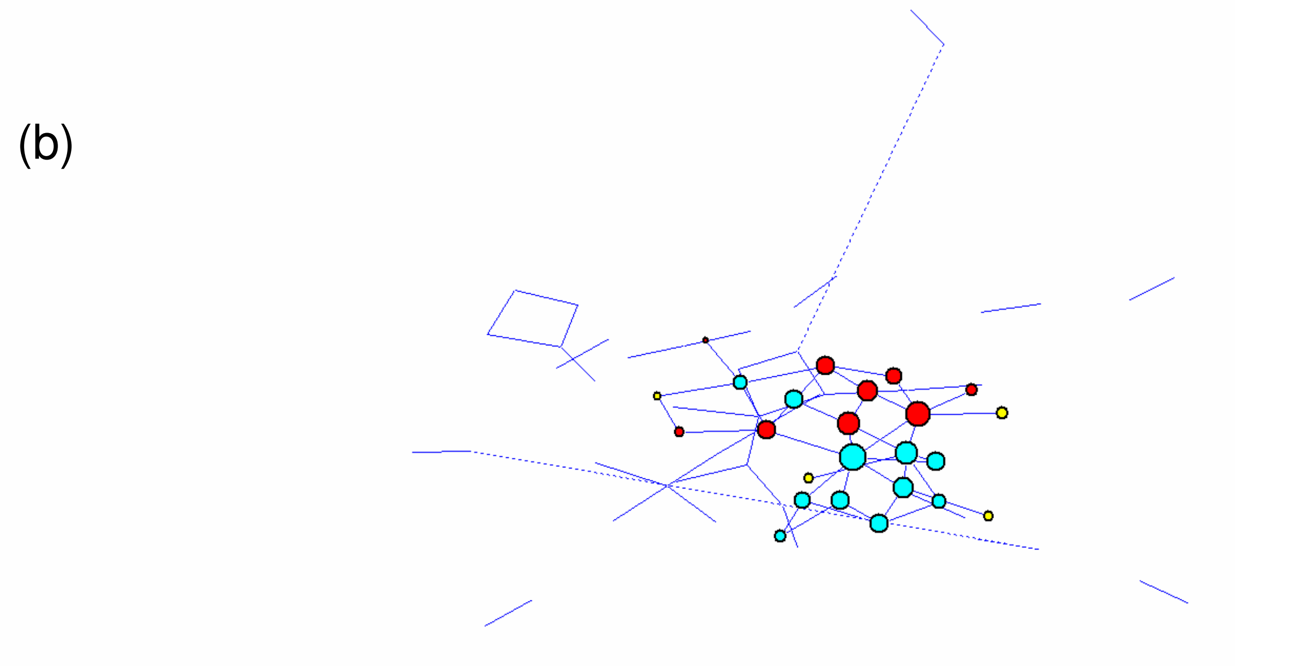

Supplement: Supplementary file 3 [file tpj0062-1058-SD3.pdf]
