# Supplementary material for: Metabolic profiling reveals local and systemic responses of host plants to nematode parasitism
Source: Plant J. 2010 May 11;62(6):1058–71. doi: 10.1111/j.1365-313X.2010.04217.x (PMC2904900; doi:10.1111/j.1365-313X.2010.04217.x)

Figure S4. Difference of the number of significant correlations (degree) of single metabolites for the local response (a) syncytium versus c-roots and the systemic response (b) i-shoots versus c-shoots. (Pearson's correlation analysis, $p<0.05$, correlation coefficient $-0.7< r >0.7$)

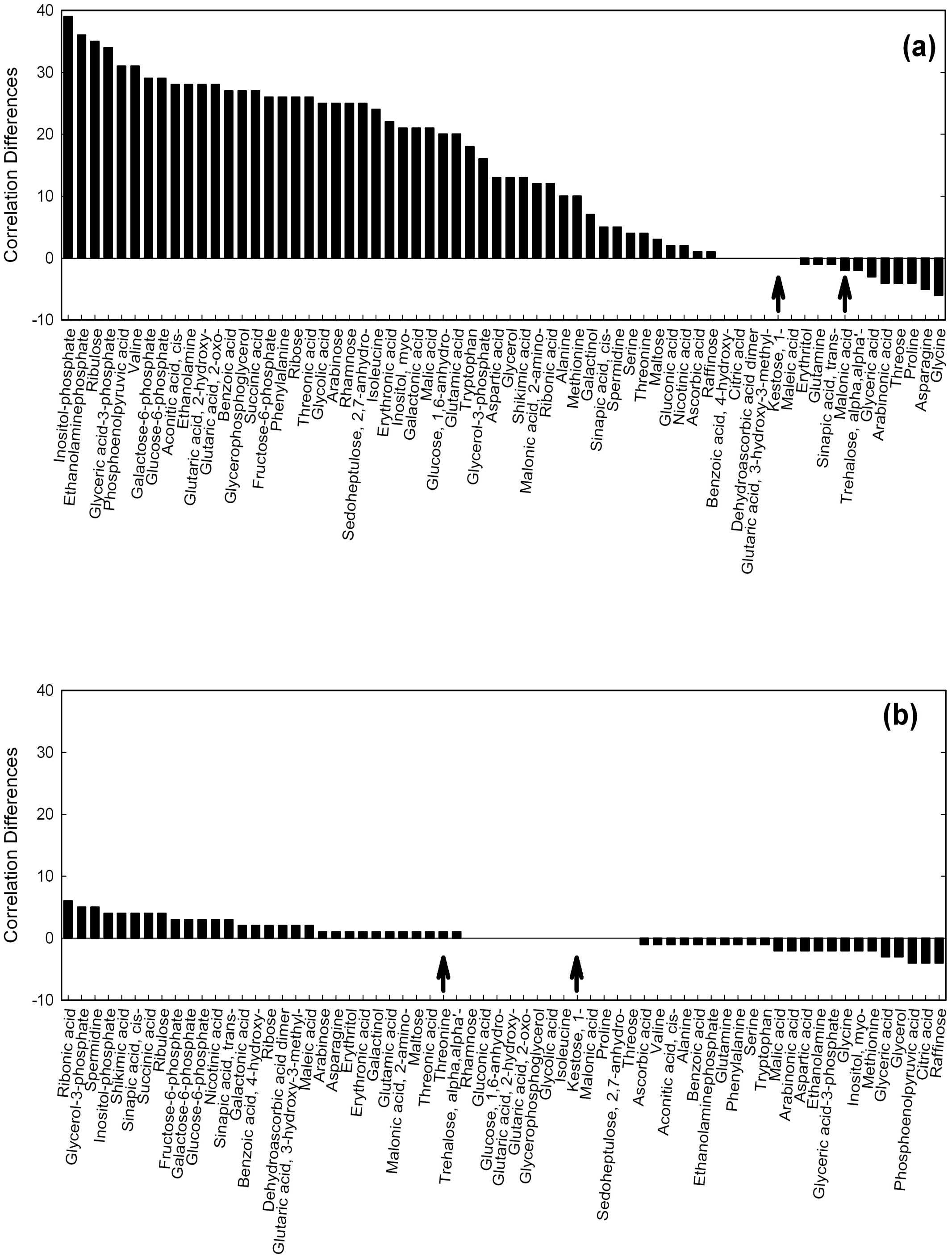

Supplement: Supplementary file 4 [file tpj0062-1058-SD4.pdf]
